# Melanonychia and skin hyperpigmentation with hydroxyurea therapy

**DOI:** 10.4103/0253-7613.62394

**Published:** 2010-02

**Authors:** Sankha Koley, Sanjiv Choudhary, Atul Salodkar

**Affiliations:** Department of Dermatology, Bankura Sammelani Medical College, West Bengal, India. E-mail: skoley@gmail.com; 1 Department of Dermatology, J.N.M.C. Sawangi, Wardha, Maharastra, India

Sir,

Hydroxyurea or hydroxycarbamide is an antineoplastic drug used in myeloproliferative disorders (chronic myeloid leukemia, polycythemia vera, and essential thrombocythemia), psoriasis (second-line systemic agent), and AIDS (antiretroviral properties potentiate the activity of the nucleoside reverse transcriptase inhibitor didanosine). The adverse mucocutaneous effects include hyperpigmentation, alopecia, leg ulcers, and lichenoid eruptions. We report a patient who developed hyperpigmentation of the skin and nails 10 weeks after the start of hydroxyurea therapy.

**Figure 1:**
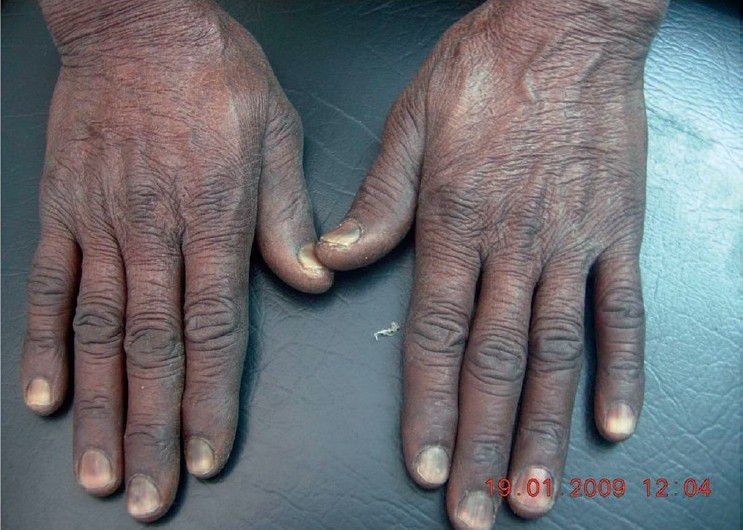
Hyperpigmentation involving hands and all 10 finger-nails; involving proximal half to two-third of the nail plates.

A 31-year-old man presented to the dermatology outpatient department with gradually progressive pigmentation of hands, legs, and all finger-nails since 1 month. The pigmentation started from proximal parts of nail plates and progressed distally. The patient was receiving hydroxyurea for chronic myeloid leukemia in a dose of 1 g/day for last three and half months. On examination, diffuse uniform hyperpigmentation was noted on all 10 finger-nails involving approximately half to two-thirds of the nail plates [Figure 1]. Hyperpigmentation was seen on hands and legs with sparing of toe nails. All routine investigations were normal except for mild anemia (hemoglobin 12.2 g/dL and hematocrit 43.8%). All necessary investigations were carried out to eliminate Vitamin B-12 deficiency, hyperbilirubinemia, Addison's disease, Cushing's syndrome, hyperthyroidism, hemosiderosis, scleroderma, and HIV. At the time of presentation, he was not taking any other drug that could have caused the nail hyperpigmentation. We considered temporary discontinuation of the drug and restart it to observe the outcome. However, the patient was lost to follow-up. According to causality assessment by Naranjo's algorithm, this event could be defined as “probable”.[1]

Nail grows at a rate of 0.1 mm/day. It takes approximately 100–120 days for the whole nail to grow. Finger nails grow faster than the toe nails. Nail changes as a result of antineoplastic drugs are asymptomatic and entirely reversible within few months after withdrawal of the offending agents. The most frequent presentation of chromonychia (various patterns of nail discoloration) induced by antineoplastic drugs is melanonychia, a dark pigmentation of nails seen in diffuse, transverse, or longitudinal band patterns. It may co-exist with diffuse pigmentation of skin, also known as melanoderma. The mechanism of hydroxyurea-induced melanonychia is still unknown; the potential causes include toxicity affecting the nail bed or nail matrix, focal stimulation of nail-matrix, melanocytes, and photosensitization. The differential diagnosis includes subungual melanoma, pigmented squamous-cell carcinoma, subungual hematoma, nevus, and hyperpigmentation owing to other drugs, including cyclophosphamide, doxorubicin, minocycline, and zidovudine.

Aste *et al.* reported nail hyperpigmentation in nine patients appearing between 6 and 24 months of hydroxyurea-therapy, the commonest presentation being longitudinal melanonychia.[2] Longitudinal melanonychia, diffuse melanonychia affecting all nails and hyperpigmentation of the skin were observed in one patient. However in our case, presentation was noted early; within 10 weeks of starting of therapy. There are reports of progressive transverse melanonychia of all 20 nails within 7 weeks of starting hydroxyurea in a patient of thrombocytosis.[3] Gropper et al. and Issaivanan et al. reported melanonychia of all 20 nails with involvement of all three mucocutaneous areas (skin, nails, and mucosa) in a 63-year-old black woman and a 10-year-old boy, respectively, within 3 months of therapy.[4,5] A dermatomyositis-like eruption was observed in two reported cases.[6] Jeevankumar et al. reported a rare case of hydroxyurea-induced blue lunula.[7] Sometimes both longitudinal melanonychia and periungual hyperpigmentation may be observed together.[8] This presentation may be confused with Hutchinson's sign in malignant melanoma. Ideally, all cases of melanonychia should be distinguished from subungual malignant melanoma.

We, therefore, report this interesting case where progressive diffuse melanonychia was observed in all 10 finger-nails with noticeable sparing of the toe nails, associated with pigmentation of hands and legs 10 weeks after starting hydroxyurea therapy.
